# Euglycemic Diabetic Ketoacidosis Secondary to Dapagliflozin in a Patient with Colon Malignancy

**DOI:** 10.1155/2019/3901741

**Published:** 2019-05-06

**Authors:** Eleni Papadokostaki, Evangelos Liberopoulos

**Affiliations:** ^1^Emergency Department, General Hospital of Heraklion “Venizeleio-Pananeio,” Heraklion, 71409, Greece; ^2^Department of Internal Medicine, Faculty of Medicine, University of Ioannina, Ioannina, 45110, Greece

## Abstract

The use of sodium-glucose cotransporter 2 (SGLT2) inhibitors for the treatment of type 2 diabetes is steadily increasing. SGLT2 inhibitors are associated with weight loss, lowering of blood pressure, and low hypoglycemia risk along with beneficial cardiovascular and renoprotective effects. In view of the increasing use of SGLT2i, physicians must be aware of their adverse effects. Euglycemic diabetic ketoacidosis (euDKA) is a well-recognized adverse effect of SGLT2i. We present here a case of euglycemic diabetic ketoacidosis secondary to dapagliflozin use in a type 2 diabetic patient with colon cancer. To the best of our knowledge, this is first report of SGLT2 inhibitor-associated euDKA in a patient with underlying colon cancer.

## 1. Introduction

Diabetic ketoacidosis (DKA) is a serious acute metabolic complication of diabetes characterized by the triad of hyperglycemia (plasma glucose >250 mg/dL), metabolic acidosis (arterial pH <7.3 and serum bicarbonate <18 mEq/L), and ketosis. It usually affects people with type 1 diabetes, but type 2 diabetics are also at risk during acute illness, such as infection, trauma, or surgery [1]. Uncommonly, patients present with plasma glucose levels of less than 250 mg/dL, which is generally defined as euglycemic DKA (euDKA) [2]. Possible precipitating factors of euDKA include the recent use of insulin, decreased caloric intake, heavy alcohol consumption, chronic liver disease, and glycogen storage disorders [3]. Munro et al. first described euDKA in patients with type 1 diabetes in ketogenic stress who were vomiting or had reduced carbohydrate intake but continued to take insulin prior to presentation, as well as in pregnancy [2]. Of note, one study found no association between severity of hyperglycemia and metabolic acidosis in DKA [4].

euDKA is a well-recognized adverse effect of sodium-glucose cotransporter 2 (SGLT2) inhibitors, the newest drugs for the treatment of diabetes. Absence of hyperglycemia can delay diagnosis and treatment, thus requiring high clinical suspicion. Since their approval many cases of euDKA have been reported, prompting the FDA to issue a warning in May 2015 [5]. Here we report a type 2 diabetic patient who presented to the emergency department with euDKA secondary to dapagliflozin use.

## 2. Case Presentation

A 64-year-old-man presented to the Emergency Department of Venizeleio Hospital, Heraklion, Crete, with a 3-day history of nausea, vomiting, and abdominal pain. His medical history included type 2 diabetes and hypertension. He had had type 2 diabetes for 10 years and was being treated at the time with vildagliptin, metformin, and dapagliflozin. Dapagliflozin was added 8 months prior to admission. He reported a weight loss of 3 Kg following the commencing of dapagliflozin, but his weight appeared stable during the last 3 months. The A1C was 7.1% one month before admission. The patient noted that he suffered from recurrent episodes of abdominal pain the last 2 months.

At presentation the blood pressure was 130/80 mmHg, heart rate 95 beats/min, temperature 36.8°C, and oxygen saturation 98% on ambient air. The patient appeared mildly dehydrated with a BMI of 26.5 kg/m^2^. The abdomen was soft with mild tenderness in the epigastrium and left upper quadrant. The remainder of the physical examination was normal.

An arterial blood gas analysis was performed and revealed metabolic acidosis pH 7.33, HCO_3_^−^ 10.9 mEq/L, and PCO_2_ 21 mmHg with an increased anion gap at 29 mmol/L. Plasma glucose was mildly elevated at 203 mg/dL. Serum lactate was 1.1 mmol/L, i.e., within normal range. The rest of laboratory investigation was as follows: white blood cells 7860/*μ*L, hemoglobin 14.8 g/dL, serum urea 84 mg/dL, serum creatinine 1.33 mg/dL, Na^+^ 134 mmol/L, K^+^ 4.6 mmol/L, and Cl^−^ 94 mmol/L. C-reactive protein level was moderately elevated at 8 mg/dL. Urinalysis showed glycosuria and ketonuria (glucose 4+, Oxone 4+). Measurement of serum ketones was not available.

The patient was diagnosed with euDKA possibly related to dapagliflozin use and was treated with intravenous fluids and insulin, with subsequent improvement of acid base disorders within the first 48 hours. He was also treated empirically with broad spectrum antibiotics. A computed tomography (CT) scan of the abdomen/pelvis with IV contrast was ordered to further evaluate the cause of the abdominal pain. The CT scan revealed ascending and transverse colonic dilatation proximal to a transition point in the splenic flexure and decompressed bowel distal to the obstruction, highly suspicious of colonic malignancy. Colonoscopy was then performed which showed splenic flexure cancer. The patient was transferred to the surgical clinic and underwent left hemicolectomy. He was discharged from the surgical clinic on the 9th postoperative day in good condition. Dapagliflozin was discontinued and basal-bolus insulin treatment was prescribed.

## 3. Discussion

SGLT2 inhibitors are the newest drugs for the treatment of type 2 diabetes. They act by preventing glucose reabsorption in the renal tubule, thus promoting glucosuria [6]. Their mechanism of action is independent of insulin, thus allowing their use in any stage of type 2 diabetes. Apart from their glucose-lowering action they exert some beneficial effects that make them very appealing in the treatment of diabetes. SGLT2 inhibitors are associated with a low incidence of hypoglycemia and have a favorable effect on body weight and blood pressure [7]. Moreover, cardiovascular outcomes trials (CVOTs) of empagliflozin, canagliflozin, and dapagliflozin demonstrated reduced atherosclerotic cardiovascular events in patients with established cardiovascular disease [8]. They have also showed benefits on reducing hospitalization for heart failure and progression of renal disease regardless of established atherosclerotic cardiovascular disease or history of heart failure [8]. Based on these findings American and European guidelines recommend SGLT2 inhibitors (or GLP-1 receptor agonists) with proven cardiovascular benefit for patients with type 2 diabetes and atherosclerotic cardiovascular disease (ASCVD) [9]. SGLT2 inhibitors are also recommended for patients with ASCVD in whom heart failure predominates as well as patients with chronic kidney disease [9]. SGLT2 inhibitors are also a preferable choice when weight loss is the main concern in diabetes treatment [9].

Adverse effects associated with SGLT2 inhibitors include mycotic genital infections (mostly vaginitis in women, balanitis in men) and euDKA [10]. SGLT2 inhibitors have also been associated with an increased risk of acute kidney injury, dehydration, and orthostatic hypotension [10]. Canagliflozin has been linked to lower-extremity amputations and bone fractures in some, but not all, studies [11, 12].

The mechanisms of SGLT2 inhibitor-associated euDKA are not fully elucidated. These agents decrease fasting and postprandial plasma glucose levels, which in turn decrease insulin secretion [13]. In contrast, glucagon secretion is increased as a result of diminished paracrine inhibition by insulin and the direct effect of SGLT2 inhibitors on pancreatic a-cells [14]. The lower insulin-to-glucagon ratio coupled with markedly elevated urinary glucose clearance favors ketogenesis [15]. Moreover, SGLT2 inhibitors are believed to reduce clearance of ketones by the kidneys [16]. Surprisingly, mild hyperketonemia has been proposed as one of the mechanisms responsible for the cardioprotection in the EMPA-REG OUTCOME trial [17].

Not all patients treated with SGLT2 inhibitors are susceptible to developing euDKA. Most patients with SGLT2 inhibitor-associated euDKA have type 1 diabetes or latent autoimmune diabetes in adults (LADA) or have long-standing insulin deficient type 2 diabetes and develop DKA in the presence of stress and illness [18]. In the case series from canagliflozin clinical trials, 6 of 12 patients had low C-peptide levels (≤0.51 ng/mL) and/or were positive for GAD65 antibodies [19]. Indeed, many cases of SGLT2 inhibitor-associated euDKA have occurred in individuals with presumed type 2 diabetes who were subsequently diagnosed with LADA and were positive for GAD65 antibodies [18]. Patients with type 1 diabetes have a higher risk of euDKA than those with type 2 diabetes and currently SGLT2 inhibitors are not approved for the treatment of type 1 diabetes. However, SGLT2 inhibitors have been investigated as an adjunctive therapy to insulin for patients with type 1 diabetes in clinical trials [20–22]. These trials have demonstrated increases in the absolute risk of DKA, which appear to be dose-dependent. In the EASE trials, empagliflozin improved glycemic control and weight in type 1 diabetes but increased the risk of ketoacidosis at the doses of 10 and 25 mg [22]. An international consensus on risk management of diabetic ketoacidosis in patients with type 1 diabetes treated with SGLT inhibitors has been recently published, providing data regarding the safety of SGLT inhibitors in type 1 diabetes [23].

Common precipitating factors for SGLT2 inhibitor-associated euDKA include abrupt insulin dose reduction, low caloric and fluid intake, acute illness (e.g., infection, gastroenteritis, myocardial infarction, and stroke), major surgical procedures, bariatric surgery, low carbohydrate diets, excessive alcohol intake, and prolonged starvation [24]. Under such stressful conditions, metabolism shifts from carbohydrate to fat oxidation, thereby predisposing patients treated with SGLT2 inhibitors to developing ketonemia and euDKA.

An analysis of case reports of SGLT2 inhibitor-related DKA from the FDA adverse event reporting system found an extremely wide range of age and body weight and a highly variable duration of SGLT2 inhibitor treatment before onset of DKA [25]. These data suggest that SGLT2 inhibitor-associated euDKA may not be limited to any particular demographic or comorbid subpopulation and can occur at any time of SGLT2 inhibitor use [25].

As already noted, euDKA also occurs in diabetic patients, both type 1 and type 2, who are not taking SGLT2 inhibitors. Therefore, when a patient taking an SGLT2 inhibitor presents with euDKA it cannot be known with certainty that the SGLT2 inhibitor caused euDKA in that individual. Although case reports are frequently cited to emphasize the euDKA risk with SGLT2 inhibitors, the baseline incidence of euDKA should be taken into account. Thus, true incidence in real clinical practice remains unknown.

The incidence of euDKA in randomized controlled trials of SGLT2 inhibitors with T2D is low. In the CANVAS trial a small number of events of diabetic ketoacidosis were observed with canagliflozin and placebo (0.6 vs. 0.3 participants with an event per 1000 patient years; hazard ratio, 2.33; 95% CI, 0.76 to 7.17) [11]. In EMPA-REG OUTCOME with approximately 7,000 patients, the frequency of reported events of DKA was less than 0.1% [26]. In DECLARE-TIMI 58, DKA was more common in the dapagliflozin than in placebo group (0.3% vs. 0.1%; hazard ratio, 2.18; 95% CI, 1.10 to 4.30) [27]. Data from 2 cohort studies indicate increased risk of DKA after initiation of SGLT2 inhibitors [10, 28]. A register-based cohort study from 2 countries, Sweden and Denmark, from July 2013 to December 2016, compared 17,213 new users of SGLT2 inhibitors with 17,213 new users of glucagon-like peptide 1 (GLP1) receptor agonists [10]. Use of SGLT2 inhibitors, as compared with GLP1 receptor agonists, was associated with a 2-fold increased risk of DKA [10]. A cohort study in the USA compared diabetic individuals who had newly started treatment with either an SGLT2 inhibitor or a dipeptidyl peptidase-4 (DPP4) inhibitor between April 1, 2013, and December 31, 2014. The unadjusted rate of DKA within 180 days after the initiation of an SGLT2 inhibitor was about twice the rate after the initiation of a DPP4 inhibitor before propensity-score matching to adjust for differences in individual characteristics (4.9 vs. 2.3 events per 1000 person years; HR, 2.1 (95% CI, 1.5, 2.9)). After propensity matching the hazard ratio was 2.2 (95% CI, 1.4, 3.6) [28].

The American Association of Clinical Endocrinologists and American College of Endocrinology in order to minimize the risk of SGLT2 inhibitor-associated DKA recommend stopping the SGLT2 inhibitor at least 24 hours prior to elective surgery, planned invasive procedures, or severe stressful physical activity; avoidance of abrupt insulin withdrawal or excessive dose decreases; and avoidance of excessive alcohol intake and/or very low carbohydrate or ketogenic diets [18]. Routine measurement of urine ketones is not recommended during use of SGLT2 inhibitors and can be confusing. This is because the nitroprusside reaction, which is a widely available method used to measure ketones in the urine, does not detect *β*-hydroxybutyrate, the predominant ketone product in ketoacidosis [1, 18]. Indeed, some investigators have recommended the use of serum *β*-hydroxybutyrate for diagnosis and monitoring of DKA [29]. Goldenberg et al. have also provided recommendations for prevention and diagnosis of SGLT2 inhibitor-related DKA. All patients should be educated on the signs and symptoms of DKA prior to starting an SGLT2 inhibitor. They should also be provided with clear instructions to seek medical help when they are experiencing symptoms suggestive of DKA, such as abdominal pain, nausea, vomiting, and fatigue [30].

In our patient euDKA probably developed due to acute illness because of the underlying colon malignancy and large bowel dilatation. The recurrent episodes of abdominal pain required further evaluation and led to colon malignancy diagnosis. To the best of our knowledge, this is first report of SGLT2 inhibitor-associated euDKA in a patient with underlying colon cancer. Further studies are needed to investigate whether malignancy increases the risk of euDKA when using an SGLT2 inhibitor.

## 4. Conclusion

SGLT2 inhibitors are generally well tolerated and make an appealing option because of their cardiovascular, renoprotective, and other benefits. euDKA is a possible adverse effect of SGLT2 inhibitors. It is important for clinicians to identify patients at risk of euDKA and provide relevant advice. A search for underlying malignancy may be warranted when no other risk factor is recognized.
